# Comparative analysis of the distribution and antifungal susceptibility of yeast species in cat facial hair and human nails

**DOI:** 10.1038/s41598-024-65730-w

**Published:** 2024-06-26

**Authors:** Chompoonek Yurayart, Sara Niae, Orawan Limsivilai, Naris Thengchaisri, Panpicha Sattasathuchana

**Affiliations:** 1 Department of Microbiology and Immunology, Faculty of Veterinary Medicine, Kasetsart University, Bangkok, 10900 Thailand; 2 Faculty of Veterinary Science, Rajamangala University of Technology Srivijaya, Nakhon Si Thammarat, 80240 Thailand; 3 Department of Companion Animal Clinical Sciences, Faculty of Veterinary Medicine, Kasetsart University, 50 Ngamwongwan Rd, Latyao, Jatujak, Bangkok, 10900 Thailand

**Keywords:** Antifungal susceptibility, Cats, Humans, Fungal infection, Zoonosis, Fungi, Epidemiology

## Abstract

Zoonotic yeast species have been implicated in disease development in both humans and cats. This study analyzed the yeast mycobiota present in feline facial hair and human nails and explored potential interspecies associations. A total of 118 biological specimens were examined, including 59 feline facial hair and 59 human nail samples. DNA extraction and DNA sequencing were performed to identify the specific yeast species. The most predominant yeast species in humans and cats were selected for antifungal susceptibility testing (itraconazole, ketoconazole, miconazole, and terbinafine). The findings unveiled diverse yeast species in cats and humans. *Malassezia pachydermatis* (45.8%) and *Malassezia furfur* (30.5%) were the most common yeast species in cats and humans, respectively. However, no significant correlation was detected between the yeast species identified in cats and their owners residing in the same household (*p* > 0.05). Miconazole exhibited the highest minimum inhibitory concentrations (MICs) against *Malassezia pachydermatis* and *Malassezia furfur* in both cat and human isolates, whereas terbinafine showed the lowest MICs against most *Malassezia pachydermatis* and *Malassezia furfur* in both cat and human isolates. Diverse yeast species in cat facial hair and human nails suggest possible cross-contamination among humans, pets, and environments.

## Introduction

Yeast, a unicellular fungus, includes recognized potential zoonotic organisms such as *Candida* spp., *Cryptococcus* spp., *Malassezia* spp., *Rhodotorula* spp., and *Trichosporon* spp.^1–4^. These organisms can cause various human and animal diseases^2,4^. The diseases related to yeast infections can range from minor skin diseases to systemic infections, for instance, pityriasis versicolor, seborrheic dermatitis, atopic eczema, and folliculitis^2,5,6^. The most identified yeast organisms in the skin of humans and cats are *Malassezia furfur* and *Malassezia pachydermatis*, respectively^2,6–8^. In healthy hosts, both species may not induce any clinical diseases. However, these organisms can overgrow in immunocompromised patients, leading to local skin diseases and systemic infections^2,5,7^. Lipid-dependent *Malassezia* spp. and *Malassezia pachydermatis* can be transmitted between humans and cats through direct or indirect skin contact^9^.

Previous studies have reported the transmission of pathogens between cats and humans sharing the same living spaces^9–11^. One of the most common interactions between humans and their cats is petting^12,13^. The bonding between humans and cats is increased by petting activity^12,14^. The temporal and chin regions of cats are commonly touched by their human owner^12^, posing a potential risk of disease transmission during these petting activities and through cohabitation. To the authors’ knowledge, there have been no reports on the association between the presence of yeast organisms in humans and in cats in the same household. Therefore, the objectives of this study were (1) to identify the yeast organisms in humans and cats, (2) to evaluate the yeast organism distribution in humans and cats who are sharing the same household, and (3) to evaluate the in vitro efficacy of antifungal drug susceptibility against the most common yeast organisms in cats and humans.

## Materials and methods

### Ethical considerations for humans and animals

The human research protocol and animal use protocol for this study were submitted to and reviewed by the Kasetsart University Research Ethics Committee (COE#66/014) and the Kasetsart University Institutional Animal Care and Use Committee (ID#ACKU66-VET-041), respectively. The informed consent was obtained from all subjects and/or their guardian (s). The cats' owners also read and signed the consent form for the study on their pet cats. All methods were performed in accordance with the relevant guidelines and regulations. The study was conducted in compliance with the Animal Research: Reporting of In Vivo Experiments (ARRIVE) guidelines.

### Humans and animals

This was a prospective cross-sectional study. A total of 118 samples were collected from 59 pairs of owners and their healthy cats. Sample collection was performed at the Kasetsart University Veterinary Teaching Hospital, Bangkok, Thailand. All owners completed a questionnaire. Owners collected their nail samples by trimming their own fingernails. Data on age, sex, breed, body weight, body condition score, and hair length (shorthair or longhair) were recorded for each cat. A general physical examination and complete blood count (Sysmex XN-1000TM Hematology Analyzer, Sysmex, IL, USA) were performed to evaluate the cats’ health condition. All cats were evaluated for the following biochemistry parameters (IL Lab 650 chemistry system, Diamond Diagnostics, MA, USA): serum creatinine, blood urea nitrogen, alkaline phosphatase, protein, globulin, and albumin. During the examination, facial hair and scales were collected from cats by a veterinarian using a sterile brush. The brushing technique involved brushing each of the following areas three times: from the end of the nostril to the forehead, from the forehead to the occipital area, from the lip to the mandibular joint, and in the chin area. All cats were tested for the feline leukemia antigen and the feline immunodeficiency virus antibody using a commercially available immunochromatography rapid test (Witness®, Lyon Cedex, France).

### Yeast culture and phenotype identification

Cat facial hair samples and human nail samples were inoculated on yeast culture media, including Sabouraud dextrose agar (Difco, USA) and Leeming and Notman agar containing oxytetracycline (50 mg/L; Oxycline, Bangkok, Thailand) to facilitate yeast culture and isolation^15^. The agar plates were incubated at 30 °C and examined daily for 14 days. The colonies were macroscopically and microscopically examined, and urea hydrolysis was used to differentiate the yeast genus. Selected lipid utilization tests for identification of *Malassezia* spp., including Cremophor EL test, Tween 60-esculin agar, and the Tween assimilation test, were performed^15^. All yeast isolates were stored at − 80 °C in glycerol stocks for further in vitro antifungal susceptibility testing. Additionally, yeast cells were harvested and stored at − 20 °C for species identification through DNA barcoding.

### Molecular identification of yeast species

Representative yeast isolates of each genus and species with distinguished phenotypes were selected for species confirmation using the fungal DNA barcoding method^16^. The genomic DNA of all selected isolates was extracted using a modified version of a previously described technique^17^. In brief, the yeast pellet was frozen at − 20 °C overnight and incubated with lysis buffer (sodium dodecyl sulfate salt, 0.5 g; Himedia, Mumbai, India), NaCl (1.4 g; Carlo Erba, MI, USA), disodium ethylenediaminetetraacetic acid (0.73 g; Biobasic, Ontario, Canada), 1 M Tris–HCl (20 ml; Biobasic, Ontario, Canada), and 2-mercaptoenol (5 µl; Sigma Aldrich, Steinheim, Germany) at 65 °C for 1 h with occasional vortexing. The lysate was extracted with phenol–chloroform-isoamyl alcohol (25:24:1, vol/vol/vol), and the DNA was precipitated with cold isopropanol. The internal transcribed spacer (ITS) region of the ribosomal DNA was amplified using the primer set SR6R (5′-AAGTATAAGTCGTAACAAGG-3′) and ITS4 (5′-TCCTCCGCTTATTGATATGC-3′), and the amplicons were examined on 1.2% agarose gel electrophoresis. All polymerase chain reaction products were purified and sequenced in forward and reverse directions using ABI BigDye Terminator v.3.1 (Macrogen Inc., Korea). Sequences were bidirectionally assembled and manually corrected for consensus sequences using BioEdit v.7.2.5 (https://bioedit.software.informal.com). ITS sequence similarity and species identification were determined using the BLASTn tool from the National Center for Biotechnology Information (http://blast.ncbi.nlm.nih.gov/Blast.cgi). Gene sequences of all representative isolates were submitted to GenBank (accession numbers are shown in Supplementary Table S1).

### In vitro antifungal susceptibility profiles

*Malassezia furfur* and *Malassezia pachydermatis*, the most predominant yeast species in humans and cats, were selected for in vitro antifungal susceptibility testing. A modified microdilution broth method following the guidelines of the Clinical and Laboratory Standards Institute (CLSI M27-A3) was employed^18^. Sabouraud dextrose broth (SDB) supplemented with 1% Tween 80 (Sigma-Aldrich, MO, USA) (SDB-T_80_) was used as a diluting medium in all steps of the antifungal susceptibility testing of the *Malassezia* yeasts. Antifungal agents, including itraconazole (ITZ), ketoconazole (KZ), miconazole (MZ), and terbinafine (TERB) (all purchased from Sigma-Aldrich, MO, USA), were dissolved for stock solutions in dimethyl sulfoxide (Sigma-Aldrich, MO, USA) and diluted in SDB-T_80_ before being distributed into 96-well microdilution plates. Final concentrations ranged from 0.06 to 32 µg/ml for ITZ, KZ, and MZ and from 0.02 to 8 µg/ml for TERB. The yeast inocula were obtained from 7-day-old cultures, measured by spectrophotometry at 530 nm, and diluted in SDB-T_80_ for a final cell concentration of 1 × 10^4^–5 × 10^4^ CFU/ml. After incubating at 32 °C for 72 h, the minimum inhibitory concentrations (MICs) were determined by observation with the naked eye. *Candida parapsilosis* ATCC 22019 was included as a quality control.

### Statistical analysis

Commercially available statistical software packages (JMP version JMP Pro 10, SAS Institute, Cary, NC, USA; GraphPad Prism version 9.0, Graph-Pad Software, La Jolla, CA, USA; and STATA version 14.2 Stata Corp LLC, College Station, TX, USA) were used for statistical analyses. Results were considered statistically significant when* p* < 0.05.

Each yeast organism's prevalence on human nails and cat facial hair (and 95% confidence intervals [CI]) was determined. A normality test was performed for all data using the Shapiro–Wilk test. Fisher’s exact test was used to analyze the association between yeast organisms in cats and humans sharing the same household.

## Results

A total of 118 biological specimens, 59 cat facial hair and 59 human nail samples, were included in the study. The mean ± standard deviation (SD) age of cats was 5.7 ± 3.7 years. Of the 59 cats, there were 37 males and 22 females. The mean ± SD of the cats’ body weight was 5.1 ± 1.5 kg. Domestic Shorthair (n = 27) was the most common breed, followed by Persian (n = 19), Maine Coon (n = 4), Mixed Long-Haired (n = 3), Bengal (n = 2), Scottish Fold (n = 2), American Wirehair (n = 1), and Munchkin (n = 1). Fifty-two cats were strictly raised indoors, and seven cats had outdoor access. The mean ± SD age of the cat owners was 39.2 ± 11.5 years. There were 45 male and 14 female cat owners. The demographic characteristics of cats and cat owners are provided in Supplementary Table S2.

The cats and cat owners in the study were positive for various species of yeast. Nineteen yeast species were isolated from cat facial hair samples, as shown in Table 1. The three most common yeast species in cat facial hair samples were *Malassezia pachydermatis* (*n* = 27; 45.8%), *Malassezia nana* (*n* = 11; 18.6%), and *Malassezia furfur* (*n* = 7; 11.9%). Seventeen yeast species were isolated from human nails. The most frequently identified yeast species in human nails were *Malassezia furfur* (*n* = 18, 30.5%), *Candida parapsilosis* (*n* = 12, 20.3%), *Malassezia sympodialis* (*n* = 6, 10.2%), and *Trichosporon asahii* (*n* = 6, 10.2%). The prevalence and 95% CI of isolated yeast species for each host are shown in Table 1.

**Table 1:** Prevalence (95% CI) of identified yeast species in cat facial hair and human nails.

Identified yeast genus	Number of isolates from cats		Number of isolates from humans	
	Positive culture	Prevalence (%; 95% CI)	Positive culture	Prevalence (%; 95% CI)
*Malassezia pachydermatis*	27	45.8 (32.7–59.3)	2	3.4 (0.4–11.7)
*Malassezia furfur*	7	11.9 (4.9–22.9)	18	30.5 (19.2–43.9)
*Malassezia nana*	11	18.6 (10.0–30.9)	0	0
*Malassezia sympodialis*	0	0	6	10.2 (3.8–20.8)
*Malassezia cuniculi*	1	1.7 (0.0–9.1)	0	0
*Malassezia japonica*	1	1.7 (0.0–9.1)	0	0
*Candida parapsilosis*	1	1.7 (0.0–9.1)	12	20.3 (11–32.8)
*Candida ciferrii*	1	1.7 (0.0–9.1)	0	0
*Candida glabrata*	0	0	1	1.7 (0.0–9.1)
*Candida orthopsilosis*	0	0	2	3.4 (0.4–11.7)
*Candida albicans*	0	0	3	5.1 (1.1–14.2)
*Candida tropicalis*	0	0	2	3.4 (0.4–11.7)
*Candida metapsilosis*	0	0	1	1.7 (0.0–9.1)
*Candida fermentati*	0	0	1	1.7 (0.0–9.1)
*Candida krusei*	0	0	1	1.7 (0.0–9.1)
*Aureobasidium melanogenum*	5	8.5 (2.8–18.7)	0	0
*Trichosporon asahii*	2	3.4 (0.4–11.7)	6	10.2 (3.8–20.8)
*Trichosporon insectorum*	1	1.7 (0.0–9.1)	0	0
*Rhodotorula mucilaginosa*	1	1.7 (0.0–9.1)	0	0
*Rhodotorula toruloides*	1	1.7 (0.0–9.1)	0	0
*Rhodotorula kratochvilovae*	1	1.7 (0.0–9.1)	0	0
*Coniochaeta rhopalochaeta*	1	1.7 (0.0–9.1)	0	0
*Debaryomyces nepalensis*	1	1.7 (0.0–9.1)	0	0
*Naganishia diffluens*	1	1.7 (0.0–9.1)	0	0
*Saccharomyces cerevisiae*	1	1.7 (0.0–9.1)	0	0
*Sympodiomycopsis* sp.	1	1.7 (0.0–9.1)	0	0
*Diutina rugosa*	0	0	1	1.7 (0.0–9.1)
*Exophiala dermatitidis*	0	0	1	1.7 (0.0–9.1)
*Fereydounia khargensis*	0	0	1	1.7 (0.0–9.1)
*Meyerozyma guilliermondii*	0	0	1	1.7 (0.0–9.1)
*Yarrowia lipolytica*	0	0	1	1.7 (0.0–9.1)

*CI* confidence interval.

Among the 31 identified yeast species in the present study, three (*Malassezia pachydermatis*, *Malassezia furfur*, and *Candida parapsilosis*) were recovered from cats and owners in the same household (Table 2). There was no association between *Malassezia pachydermatis* (*p* = 0.326), *Malassezia furfur* (*p* = 0.184), or *Candida parapsilosis* (*p* = 0.203) among cats and humans in the same household.

**Table 2:** Association between isolated yeast species collected from cat facial hair and human nails.

Recovered yeast species	Isolates from cats	Isolates from humans		*p* value
		Positive	Negative	
*Malassezia pachydermatis*	Positive	2	25	0.326
	Negative	0	32	
*Malassezia furfur*	Positive	4	3	0.184
	Negative	14	38	
*Candida parapsilosis*	Positive	1	0	0.203
	Negative	11	47	

Twenty-nine isolates of *Malassezia pachydermatis* from cats (n = 27) and humans (n = 2) were tested for in vitro antifungal susceptibility. The data are presented for isolates from all 27 cats and for the matched isolates from cats and humans in the same household (Table 3). The MIC_50_ and MIC_90_ of ITZ, KZ, MZ, and TERB against *Malassezia pachydermatis* isolated from cats were 0.03 and 2 µg/ml, 0.13 and 8 µg/ml, 2 and 64 µg/ml, and 0.13 and 0.5 µg/ml, respectively. The MIC_50_ and MIC_90_ of ITZ, KZ, MZ, and TERB against *Malassezia pachydermatis* isolated from cats and humans sharing the same household (*n* = 4) were similar: 1 and 4 µg/ml for ITZ, 2 and 4 µg/ml for KZ, 32 and 64 µg/ml for MZ, and 0.06 and 0.13 µg/ml for TERB, respectively.

**Table 3:** Antifungal susceptibility of *Malassezia pachydermatis* isolates and matched yeast isolates in cats and humans from the same household.

Antifungal agent	Cats (*n* = 27)				Matched yeast isolates from cats (*n* = 2)				Matched yeast isolates from humans (*n* = 2)			
	MIC (*n*)	GM ± SD	MIC_50_	MIC_90_	MIC (*n*)	GM ± SD	MIC_50_	MIC_90_	MIC (*n*)	GM ± SD	MIC_50_	MIC_90_
Itraconazole	16 (1)	0.1 ± 3.1	0.03	2	4 (1)	2 ± 2.5	1	4	4 (1)	2 ± 2.1	1	4
	4 (1)				0.5 (1)				1 (1)			
	2 (2)											
	1 (2)											
	0.5 (2)											
	0.25 (2)											
	0.06 (3)											
	< 0.06 (14)											
Ketoconazole	> 32 (1)	0.4 ± 12.3	0.13	8	4 (2)	2.8 ± 0	2	4	4 (1)	2.8 ± 1.41	2	4
	8 (2)								1 (1)			
	4 (3)											
	2 (3)											
	0.25 (4)											
	0.13 (10)											
	0.06 (3)											
	< 0.06 (1)											
Miconazole	> 32 (5)	0.5 ± 24.9	2	64	> 32 (2)	45.3 ± 0	32	64	> 32 (1)	45.3 ± 22.6	32	64
	32 (1)								32 (1)			
	4 (2)											
	2 (10)											
	1 (4)											
	0.5 (1)											
	0.25 (1)											
	0.13 (2)											
	0.06 (1)											
Terbinafine	8 (1)	0.1 ± 3.1	0.13	0.5	0.13 (2)	0.1 ± 0	0.06	0.13	0.13 (1)	0.1 ± 0.0	0.06	0.13
	2 (1)								0.03 (1)			
	0.5 (2)											
	0.25 (6)											
	0.13 (9)											
	0.06 (3)											
	0.03(2)											
	0.02 (2)											
	< 0.02 (1)											

*GM* geometric mean, *MIC* minimum inhibitory concentration (µg/ml), *MIC*_*50*_ and *MIC*_*90*_ values that indicate 50% and 90% of isolates were inhibited, *n* number of isolates with the indicated MIC, *SD* standard deviation.

Data on the antifungal susceptibility of 25 *Malassezia furfur* isolates recovered from cats (*n* = 7) and humans (*n* = 18) are presented in Table 4. The MIC_50_ of all tested drugs against *Malassezia furfur* from cat and human isolates were similar: 0.5 µg/ml (cats) and 0.25 µg/ml (humans) for ITZ, 0.5 µg/ml (cats and humans) for KZ, 2 µg/ml (cats and humans) for MZ, and 0.13 µg/ml (cats and humans) for TERB. However, the MIC_90_ of most isolates from cats were higher than the MIC_90_ of isolates from humans sharing the same household: 8 µg/ml (cats) and 2 µg/ml (humans) for ITZ, 16 µg/ml (cats) and 2 µg/ml (humans) for KZ, 64 µg/ml (cats) and 16 µg/ml (humans) for MZ, and 2 µg/ml (cats) and 0.25 µg/ml (humans) for TERB. The MIC_50_ and MIC_90_ of ITZ, KZ, MZ, and TERB against *Malassezia furfur* isolated from cats (*n* = 4) sharing the same household were 2 and 8 µg/ml for ITZ, 0.5 and 16 µg/ml for KZ, 2 and 64 µg/ml for MZ, and 0.5 and 2 µg/ml for TERB, respectively. The MIC_50_ and MIC_90_ of ITZ, KZ, MZ, and TERB against *Malassezia furfur* isolated from humans (*n* = 4) sharing the same household were 0.25 and 0.5 µg/ml for ITZ, 0.13 and 1 µg/ml for KZ, 0.5 and 0.2 µg/ml for MZ, and 0.06–0.25 µg/ml for TERB, respectively.

**Table 4:** Antifungal susceptibility of *Malassezia furfur* and matched yeast isolates in cats and humans from the same household.

Antifungal agent	Cats (*n* = 7)				Humans (*n* = 18)				Matched yeast isolates from cats (*n* = 4)				Matched yeast isolates from humans (*n* = 4)			
	MIC (*n*)	GM ± SD	MIC_50_	MIC_90_	MIC (*n*)	GM ± SD	MIC_50_	MIC_90_	MIC (*n*)	GM ± SD	MIC_50_	MIC_90_	MIC (*n*)	GM ± SD	MIC_50_	MIC_90_
Itraconazole	8 (1)	0.7 ± 2.8	0.5	8	16 (1)	0.3 ± 3.7	0.25	2	8 (1)	1.7 ± 3.4	2	8	0.5 (1)	0.3 ± 0.2	0.25	0.5
	2 (2)				2 (1)				2 (2)				0.25 (2)			
	0.5 (1)				0.5 (2)				0.25 (1)				0.13 (1)			
	0.25 (1)				0.25 (7)											
	0.13 (2)				0.13 (6)											
					0.06 (1)											
Ketoconazole	16 (1)	0.6 ± 5.9	0.5	16	16 (1)	0.5 ± 3.7	0.5	2	16 (1)	1.6 ± 7.5	0.5	16	1 (1)	0.3 ± 0.4	0.13	1
	2 (1)				2 (2)				2 (1)				0.5 (1)			
	0.5 (2)				1 (3)				0.5 (2)				0.13 (2)			
	0.25 (1)				0.5 (4)											
	0.13(2)				0.25 (3)											
					0.13 (5)											
Miconazole	> 32 (1)	1.6 ± 23.5	2	64	16 (2)	1.4 ± 4.8	2	16	> 32 (1)	2.8 ± 31.2	2	64	2 (2)	1 ± 0.9	0.5	2
	4 (2)				4 (2)				4 (1)				0.5 (2)			
	2 (2)				2 (6)				2 (1)							
	0.13 (1)				1 (1)				0.13 (1)							
	0.06 (1)				0.5 (6)											
					0.06 (1)											
Terbinafine	2 (1)	0.2 ± 0.7	0.13	2	0.5 (1)	0.1 ± 0.1	0.13	0.25	2 (1)	0.5 ± 0.8	0.5	2	0.25 (1)	0.1 ± 0.1	0.06	0.25
	1 (1)				0.25 (6)				1 (1)				0.13 (1)			
	0.5 (1)				0.13 (4)				0.5 (1)				0.06 (1)			
	0.13 (2)				0.06 (5)				0.06 (1)				0.03 (1)			
	0.06 (1)				0.03 (1)											
					0.03 (1)											

*GM* geometric mean, *MIC* minimum inhibitory concentration (µg/ml), *MIC*_*50*_ and *MIC*_*90*_ values that indicate 50% and 90% of isolates were inhibited, *n* number of isolates with the indicated MIC, *SD* standard deviation.

## Discussion

Next-generation sequencing studies have been conducted to understand the mycobiota of animals and humans and to identify fungi associated with health conditions and disease^19,20^. However, the association between certain fungi linked to humans and animals in the same household—specifically, medically important pathogenic yeasts—has not been investigated. This study analyzed the yeast diversity in cat facial hair and human nails using a culture-dependent method, allowing the identification of certain yeast species and in vitro antifungal susceptibility testing. The analysis used 118 biological samples (facial hair from 59 cats and nails from 59 cat owners) to investigate the presence and characteristics of yeast species. The primary species of yeasts colonizing in cat facial hair and human nails were *Malassezia pachydermatis* (45.8%) and *Malassezia furfur* (30.5%), respectively. There was no association between the yeast species of cats and cat owners living in the same household.

The finding that the most prevalent yeast species in cat facial hair was *Malassezia pachydermatis* is similar to previously reported results^21^. Concerning the lipid-dependent *Malassezia* group, the predominant isolate within feline specimens was *Malassezia nana*, representing 18.6% of the isolates procured from the facial region of cats. This prevalence exceeds the documented frequency in a prior study focused on the external ear canal, where *Malassezia nana* accounted for 16.9% of isolates^22^. Furthermore, the facial hair samples from the cats in this study exhibited a lower lipid content than the samples from the external ear canal in the previous study.

According to previous research, the most abundant yeasts on the human skin are *Candida* spp.^7^. This study revealed that the predominant yeast species inhabiting human nails is *Malassezia furfur* at about 30%, which is typically a characteristic distribution on the seborrheic regions of human skin, such as the face, scalp, chest, and back. Although *Malassezia furfur* is often associated with lipid-dependent ecosystems^6,23^, its presence extends to a myriad of anatomical sites, including the nails. *Malassezia furfur* was found in 12% of cat facial hair samples; however, in previous reports, *Malassezia furfur* was identified in the external ear canal, a lipid-rich area, in only 2% of the cats studied^22^. Thus, human nails could be identified as a potential source of *Malassezia furfur* transmission to cats or vice versa.

The findings of this study revealed the diversity and potential interspecies association between yeast species in cat facial hair and cat owners’ nails. The varying yeast species in both cats and humans suggest the possibility of potential cross-species transmission in shared environments. *Malassezia pachydermatis*, *Malassezia furfur*, and *Candida parapsilosis* were identified in cats and humans living in the same household. *Malassezia* yeasts have been recognized as cryptic species, has been studied through genotyping to reveal their roles in growth, pathogenicity, and host specificity. *Malassezia furfur* strains recovered from a bloodstream infection exhibited differences in virulence and genotypes of rDNA sequences compared to superficial and healthy skin isolates^24^. Multilocus sequencing and phylogenetic analysis demonstrated high genetic variation among *Malassezia pachydermatis* isolates from domestic animals, with two delineated clades between cat and dog populations suggesting specific host adaptation^25^. Therefore, genetic characterization using multilocus phylogenetic analysis of *Malassezia furfur* and *Malassezia pachydermatis* strains from cats and humans in aspects of health and disease should be investigated to understand better their roles in inter-species transmission (zoonotic or reverse zoonotic transmission) and specific host adaptation.

**Figure 1:**
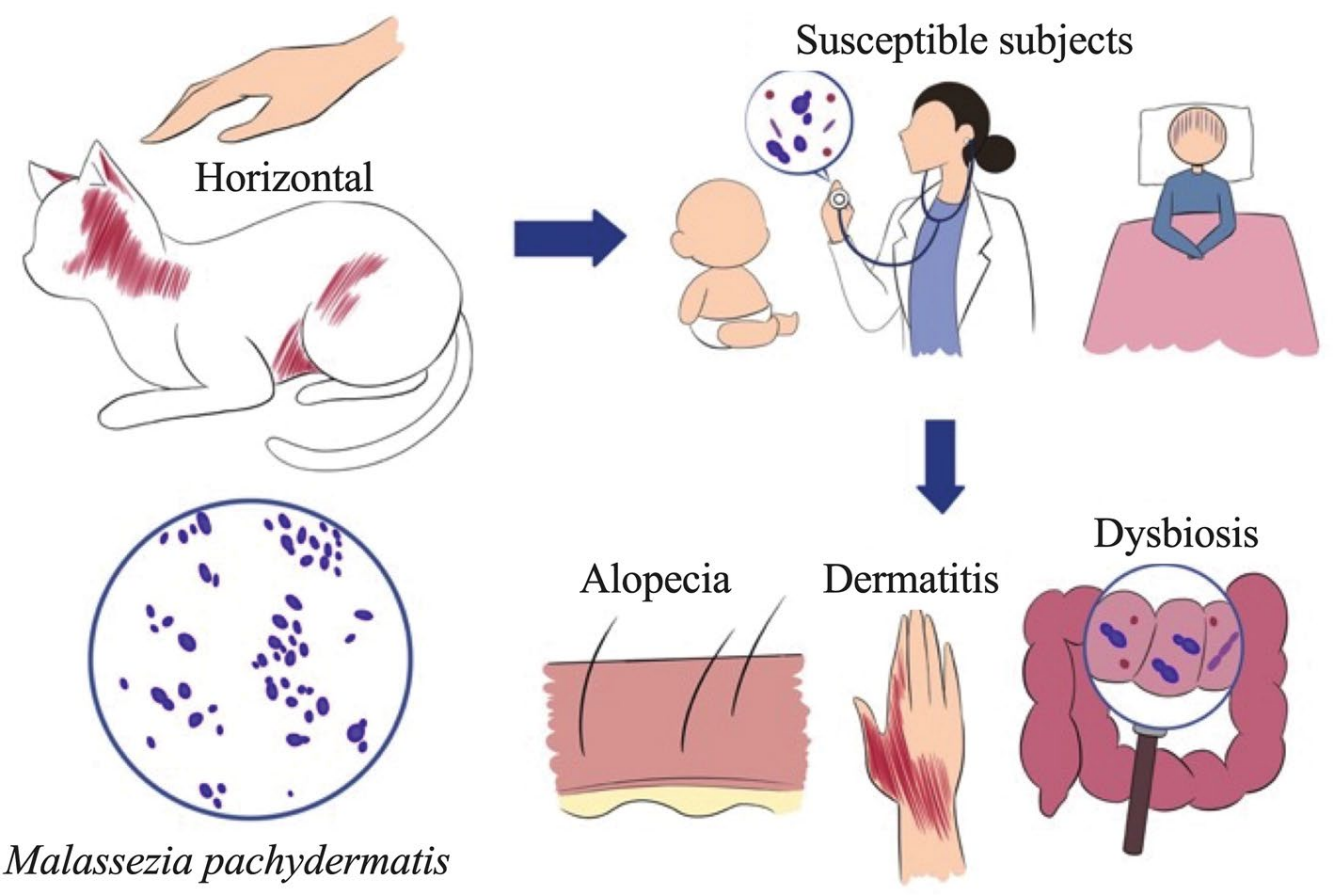
The horizontal transmission of *Malassezia pachydermatis* from cats to humans emphasizes is uncommon. This mode of transmission should be taken into consideration, particularly in the case of children and immunocompromised patients. Clinical manifestations may include dermatitis, alopecia, and dysbiosis in affected individuals.

*Malassezia pachydermatis* can be transmitted from cats to humans via direct contact and can particularly affect children and immunocompromised patients, with clinical manifestations including dermatitis, alopecia, and dysbiosis^26,27^ (Fig. 1). Although not a normal skin commensal in humans^26,27^, it can be harbored subclinically. Recently, healthcare workers have been identified as the cause of infections from pets at home to patients in hospital^28^. However, in this study, a significant correlation was not identified between yeast species in cats and their human owners. These results suggest that factors other than direct transmission within the household may contribute to the presence of these microorganisms. Such factors could include individual immune responses, variations in personal hygiene practices, or other environmental factors not explored in this study. Further studies analyzing of yeast colonization in both healthy and unhealthy cohabiting host species would enhance our understanding of yeast translocations and prevention strategies. Nonetheless, it is imperative that certain hygiene practices be implemented among health care workers with pets at home. Frequent handwashing should effectively halt the spread of *Malassezia pachydermatis*^26,29^.

In vitro antifungal drug sensitivity (ITZ, KZ, MZ, and TERB) against the most predominant yeast species, *Malassezia pachydermatis* and *Malassezia furfur*, was also evaluated. The *Malassezia pachydermatis* isolates from cats and humans sharing the same household showed the same MIC_50_ and MIC_90_ against antifungal drugs. Interestingly, the MICs of ITZ, KZ, and MZ against *Malassezia pachydermatis* from cats (*n* = 2) and humans (*n* = 2) sharing the same household were higher than the MIC_50_ of most cat isolates, whereas the MICs of TERB against *Malassezia pachydermatis* isolated from cats and humans sharing the same household were still within the level of MIC_50_ and MIC_90_ of most cat isolates. The higher MIC of antifungal drugs againts *Malassezia pachydermatis* isolates from cats and humans sharing the same household may be associated with new host adaptation by increasing the inter-host transmission and colonization abilities in multi-host species^30,31^. Further investigation on genetic fingerprints or polymorphism and virulence factors should be performed those *Malassezia pachydermatis* isolated from multi-host species in the same household.

This study is the first to report MIC values for *Malassezia furfur* in healthy cats. Isolates from cats within the same household exhibited higher MICs against all tested drugs compared to those from cat owners. This difference is possibly due to the use of antifungal shampoos or other veterinary products^21^. Veterinary products often vary in formulation, active ingredients, and concentration compared to human medicine, creating unique selective factors on the yeast populations in animals. Furthermore, the differences of frequency and method of application, microenvironment, and microbiome interactions between humans and cats, contribute to these variations^32^. Therefore, the distinct susceptibility profiles observed in *Malassezia furfur* from cats are influenced by the specific veterinary antifungal products used, leading to the development of different resistance mechanisms compared to human isolates.

The antifungal susceptibility testing procedure against *Malassezia* spp. is not yet standardized, various studies have employed the clinical and laboratory standards institute (CLSI) reference method for broth dilutional antifungal susceptibility testing with lipid-supplemented medium and inoculum size modification. Therefore, the MIC results obtained here might differ from previous studies^33^. *Malassezia pachydermatis* were exhibited higher susceptibility to all testing antifungal drugs compared to *Malassezia furfur,* showing lower geometric mean and MIC_50_ values, especially for ITZ. This finding aligned with previous studies suggesting that *Malassezia furfur* was the least susceptible species within the genus^34,35^. *Malassezia pachydermatis* isolated from cats showed high susceptibility to ITZ, KZ, and TERB based on geometric mean and MIC_50_ values. However, low-susceptibility strains of *Malassezia pachydermatis* were detected with high MICs to ITZ, KZ, MZ, and TERB. TERB is the most effective drug for *Malassezia furfur* in cats and humans, showing the lowest MIC data in our study, and is suggested as an alternative treatment for azole-unresponsive *Malassezia* infections^36^.

Among the tested antifungal agents, MZ exhibited the highest MIC against *Malassezia pachydermatis* and *Malassezia furfur* in cat and human isolates. In vitro-induced miconazole-tolerant *Malassezia pachydermatis* strains were previously performed to confirm the amino acid substitutions in the lanosterol 14-alpha-demethylase (*ERG11*) gene, which is responsible for tolerance to MZ and other azoles such as ITZ and clotrimazole. Resistance of MZ in vivo may be affected by inappropriate MZ uses, which commonly appear as active ingredients of many topical products for treating dermatitis in dogs and cats^21,37^. Therefore, further research on investigating the azole-tolerant genes related to phenotypic resistance, such as *ERG11*, should be conducted on isolates recovered from clinical strains.

One limitation of this study is that cats with dermatological lesions were not included. Cats with dermatological lesions may harbor different yeast species due to changes in mycobiota environments^18^. Long-haired cats without skin lesions frequently have dermatophyte infections on hair shafts and *Malassezia pachydermatis* infections in external ear canals^22,38^. The site of sample collection among cats with different health conditions might influence the growth and transmission of yeast. Samples from the cat’s external ear canal should also be collected and analyzed to determine the source of *Malassezia pachydermatis* translocation. Therefore, future studies should collect samples from different anatomical locations on cats and include cats with dermatological disorders or unhealthy cats. Additionally, conducting antifungal susceptibility testing on cats with dermatological disorders could provide valuable insights into antifungal selection in clinical cases for humans and cats. Another limitation is that none of the humans in this study underwent complete dermatological examinations due to restrictions on human subject research. It should be noted that the volunteers had no visible skin lesions and no history of fungal infection on their hands.

## Conclusion

The findings of this study contribute to our understanding of the mycobiota present in cat facial hair and human nails, shedding light on potential interspecies associations. Identifying diverse yeast species in both cats and humans suggests the possibility of cross-contamination or shared environmental sources and potential disease transmission to immunocompromised patients through direct contact. However, the absence of a significant correlation between specific yeast species in cats and their apparently healthy owners living in the same household indicates that transmission between these two populations may not be a significant factor. The assessment of antifungal susceptibility profiles revealed that MZ should be used cautiously.

## Completing interests

The authors declare no competing interests.

### Supplementary Information

Supplementary Information 1.

Supplementary Information 2.

## Data Availability

Correspondence and requests for materials should be addressed to PS. The data used and/or analyzed in the present study are available in the GenBank repository. The accession numbers are listed in the Supplementary Table S1. The demographic characteristics of cats and cat owners are provided in the Supplementary Table S2.
