# Supplementary material for: Comparative analysis of the distribution and antifungal susceptibility of yeast species in cat facial hair and human nails
Source: Sci Rep. 2024 Jun 26;14:14726. doi: 10.1038/s41598-024-65730-w (PMC11208614; doi:10.1038/s41598-024-65730-w)
Supplement: Supplementary file 1 — Supplementary Information 1. [file 41598_2024_65730_MOESM1_ESM.docx]

**Identified yeast genus and accession numbers**

| **Identified yeast genus** | **Accession numbers** |
| --- | --- |
| *Malassezia pachydermatis* | PP179502, PP188615, PP203075, PP203076, PP213268, PP317280, PP317279, PP317284, PP317281 |
| *Malassezia furfur* | PP125620, PP140691, PP178110, PP178111, PP179501, PP188594, PP188613, PP190928, PP203077, PP203113, PP203114, PP203115, PP204042, PP213266, PP213267, PP317282, PP317283, PP317286, PP317287, PP326112, PP333208, PP333209, PP333210, PP334787, PP922473 |
| *Malassezia nana* | PP125622, PP178112, PP178146, PP188596, PP188618, PP188634, PP188638, PP204053, PP316675, PP316684, PP916331 |
| *Malassezia sympodialis* | PP204040, PP204097, PP326157, PP326212, PP326217, PP326245 |
| *Malassezia cuniculi* | PP317285 |
| *Malassezia japonica* | PP188621 |
| *Candida parapsilosis* | PP195927, PP195928, PP204052, PP204162, PP204164, PP204171, PP204199, PP204200, PP212026, PP218380, PP218388, PP218390, PP229797, PP229796 |
| *Candida ciferrii* | PP203042 |
| *Candida glabrata* | PP218381 |
| *Candida orthopsilosis* | PP204051, PP218385 |
| *Candida albicans* | PP204170, PP204198, PP204201, PP218386 |
| *Candida tropicalis* | PP204096, PP218387 |
| *Candida metapsilosis* | PP326263 |
| *Candida fermentati* | PP326261 |
| *Candida krusei* | PP218384 |
| *Aureobasidium melanogenum* | PP195874, PP195882, PP195940, PP195943, PP195944, PP326269 |
| *Trichosporon asahii* | PP125625, PP188632, PP195871, PP204041, PP218391, PP326251, PP326252, PP326254 |
| *Trichosporon insectorum* | PP195921 |
| *Rhodotorula mucilaginosa* | PP195876 |
| *Rhodotorula toruloides* | PP195880 |
| *Rhodotorula kratochvilovae* | PP195881 |
| *Coniochaeta rhopalochaeta* | PP326268 |
| *Debaryomyces nepalensis* | PP195923 |
| *Naganishia diffluens* | PP125619 |
| *Saccharomyces cerevisiae* | PP326266 |
| *Sympodiomycopsis*sp. | PP195938 |
| *Diutina rugosa* | PP218382 |
| *Exophiala dermatitidis* | PP326265 |
| *Fereydounia khargensis* | PP333211 |
| *Meyerozyma guilliermondii* | PP218383 |
| *Yarrowia lipolytica* | PP326264 |
