# Supplementary material for: Comparative analysis of the distribution and antifungal susceptibility of yeast species in cat facial hair and human nails
Source: Sci Rep. 2024 Jun 26;14:14726. doi: 10.1038/s41598-024-65730-w (PMC11208614; doi:10.1038/s41598-024-65730-w)
Supplement: Supplementary file 2 — Supplementary Information 2. [file 41598_2024_65730_MOESM2_ESM.docx]

**Characteristics of cats and cat owners**

| **Characteristic** | **Cats** | **Cat owners** |
| --- | --- | --- |
| Number | 59 | 59 |
| Age (years) |  |  |
| Mean $($±standard deviation) | 5.7 (±3.7) | 39.2 (±11.5) |
| Sex (n; %) |  |  |
| Male | 37 (62.7) | 45 (76.3) |
| Female | 22 (37.3) | 14 (23.7) |
| Body weight (kg) |  |  |
| Mean (±standard deviation) | 5.05 (±1.5) | - |
| Breed (n; %) |  |  |
| Domestic Shorthair | 27 (45.8) | - |
| Persian | 19 (34.0) | - |
| Maine Coon | 4 (6.8) | - |
| Mixed Long-Haired | 3 (5.1) | - |
| Bengal | 2 (3.4) | - |
| Scottish Fold | 2 (3.4) | - |
| American Wirehair | 1 (1.7) | - |
| Munchkin | 1 (1.7) | - |
| Cat rearing (%; n) |  |  |
| Indoor only | 52 (88.2) | - |
| Outdoor access | 7 (11.9) | - |
